# Wastewater-Derived Microplastics as Carriers of Aromatic Organic Contaminants (AOCs): A Critical Review of Ageing, Sorption Mechanisms, and Environmental Implications

**DOI:** 10.3390/ijms262311758

**Published:** 2025-12-04

**Authors:** Zuzanna Prus, Katarzyna Styszko

**Affiliations:** 1 Department of Heat Engineering & Environment Protection, Faculty of Metals Engineering and Industrial Computer Science, AGH University of Krakow, Mickiewicza 30 Ave., 30-059 Krakow, Poland; zprus@agh.edu.pl; 2 Department of Fuel Technology, Faculty of Energy and Fuels, AGH University of Krakow, Mickiewicza 30 Ave., 30-059 Krakow, Poland

**Keywords:** microplastics, wastewater treatment, aromatic organic contaminants, sorption mechanisms, ageing processes, biofouling, environmental transport

## Abstract

Wastewater-derived microplastics (WW-MPs) are increasingly recognised as reactive vectors for aromatic organic contaminants (AOCs), yet their role in contaminant fate remains insufficiently constrained. This review synthesises current knowledge on the transformation of microplastics in wastewater treatment plants, including fragmentation, oxidative ageing, additive leaching, and biofilm formation, and links these processes to changes in sorption capacity toward phenols, PAHs and their derivatives, and organochlorine pesticides (OCPs). We summarise the dominant adsorption mechanisms-hydrophobic partitioning, π-π interactions, hydrogen bonding, and electrostatic and, in some cases, halogen bonding-and critically evaluate how wastewater-relevant parameters (pH, ionic strength, dissolved organic matter, temperature, and biofilms) can modulate these interactions. Evidence in the literature consistently shows that ageing and biofouling enhance WW-MP affinity for many AOCs, reinforcing their function as mobile carriers. However, major gaps persist, including limited data on real wastewater-aged MPs, lack of methodological standardisation, and incomplete representation of ageing, competitive sorption, and non-equilibrium diffusion in existing isotherm and kinetic models. We propose key descriptors that should be incorporated into future sorption and fate frameworks and discuss how WW-MP-AOC interactions may influence ecological exposure, bioavailability, and risk assessment. This critical analysis supports more realistic predictions of AOC behaviour in wastewater environments.

## 1. Introduction

In the 21st century, societies have become strongly dependent, and in many ways even addicted, to plastic materials in every form [1]. Extensive global use of plastic materials across all economic sectors, together with insufficient recycling rates, has led to the widespread accumulation of plastic litter in natural environments [2,3]. Particular attention is given to small polymer fragments known as microplastics (MPs), referred to as less than 5 mm in size. These include primary MPs, produced on a microscale as additives to cosmetic products or textiles, and more prevalent secondary MPs, which originate from the degradation of polymer wastes under environmental conditions, including transformations during wastewater treatment [4,5,6]. The physical presence of MPs themselves raises growing concerns about their potential impact on human health [7]. Once MPs enter the environment, they partially degrade over time and their surfaces become altered by mechanical abrasion, ultraviolet (UV) radiation, and microbial activity, thereby enhancing their pollutant-binding capacity [4]. While littering of marine shores contributes most to environmental plastic pollution, wastewater treatment plants (WWTPs) are considered a significant pathway through which altered wastewater-derived microplastics (WW-MPs), with unknown surface characteristics, are released into natural water bodies [8] or re-released into soil via fertilisers derived from treated sewage sludge [9,10]. WW-MPs can act as carriers for various co-contaminants. Owing to their hydrophobic and heterogeneous surfaces, they serve as effective adsorbates of co-occurring pollutants [11,12]. Among them, aromatic organic contaminants (AOCs) are a ring-structured subgroup increasingly detected in wastewater systems [10]. Many AOCs are mutagenic and carcinogenic, are persistent in the environment, and can bioaccumulate in living organisms [13,14]. The abundance of WW-MPs is also substantial. In a previous review, the author evaluated 53 studies covering 63 sludge samples worldwide and reported MP concentrations ranging from <1 to 240 particles/g dw (dry weight) [15]. For this reason, particular attention should be given to the sorption interactions between AOCs and WW-MPs. These interactions are environmentally relevant, as they influence the risk of AOC transfer, re-release, and substantial exposure into natural systems.

**Figure 1:**
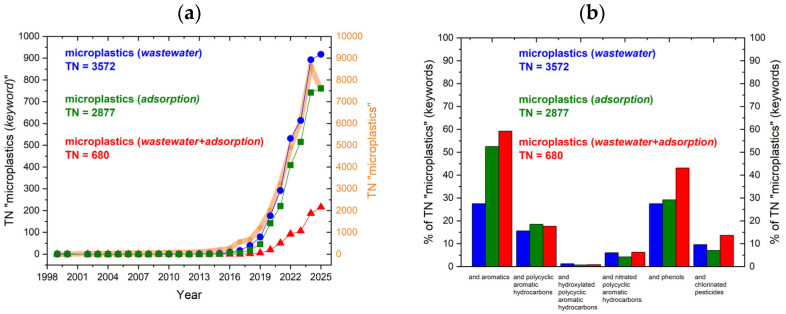
(**a**) Total number (TN) of publications containing “microplastic” (orange) and those combining it with wastewater (blue), adsorption (green), and wastewater + adsorption (red); (**b**) publications for the same keyword sets, expressed as a percentage of the TN for each group. The X-axis shows the combined search terms, and the Y-axis shows their proportional shares-data adapted from ScienceDirect.

An analysis of publication trends based on the ScienceDirect database in September 2025 revealed a marked increase in research output over the past decade (Figure 1a). To date, 37,388 articles on microplastics have been published, as shown by the broad orange line on the graph. The search term “microplastics” with the keyword “wastewater” returned 3572 articles (9.55% of TN on MP topic), with a sharp rise in the number of publications after 2018, and 917 papers published in 2025. Changing the keyword to “adsorption” resulted in 2877 articles (7.69% of TN on MP topic), with consistent growth from 2015 onward, exceeding 700 papers so far in 2025. When including both keywords, 680 articles (1.88% of TN on MP topic) were found from 2018 onward, with an upward trend, but these numbers are approximately two times lower than the previous ones.

With growing awareness of MPs’ significance in the environment, an increasing number of studies have demonstrated sorption interactions between MPs and AOCs, PAHs, OH-PAHs, N-PAHs, phenols, and organochlorinated pesticides (OCPs), as depicted in Figure 1b. The “microplastic” search phrase, combined with the wastewater keyword, accounted for approximately 28% of studies related to general aromatics; about 53% addressed aromatics; and reached 60% for wastewater and adsorption. Based on the analysis, the studied sorption interactions with MPs (red bars) can be ranked from best to worst as follows: phenols > PAHs > OCPs > N-PAHs > OH-PAHs. This distribution highlights that interactions between PAH derivatives and OCPs, as well as their fate in WW-MPs, remain insufficiently studied, despite the growing trend in general MP research.

This review examines the role of wastewater-derived microplastics (WW-MPs) as vectors of aromatic organic contaminants (AOCs), with a particular focus on phenols, polycyclic aromatic hydrocarbons (PAHs), and their hydroxylated (OH-PAHs) and nitrated (N-PAHs) derivatives, as well as organochlorinated pesticides (OCPs), which are increasingly reported in wastewater streams. The scope of this review includes the occurrence and physicochemical characteristics of WW-MPs, as well as the key sorption mechanisms governing MP-AOC complexes under the influence of environmental factors. Reviewed modelling studies in this field that rely on real, environmental MPs remain limited; therefore, sorption modelling in simplified systems under controlled conditions is often used as a proxy and is also discussed in this article. Recommendations for further improvement to modelling AOC-WW-MP sorption are discussed.

## 2. Aromatic Organic Contaminants (AOCs) in Wastewater

**Figure 2:**
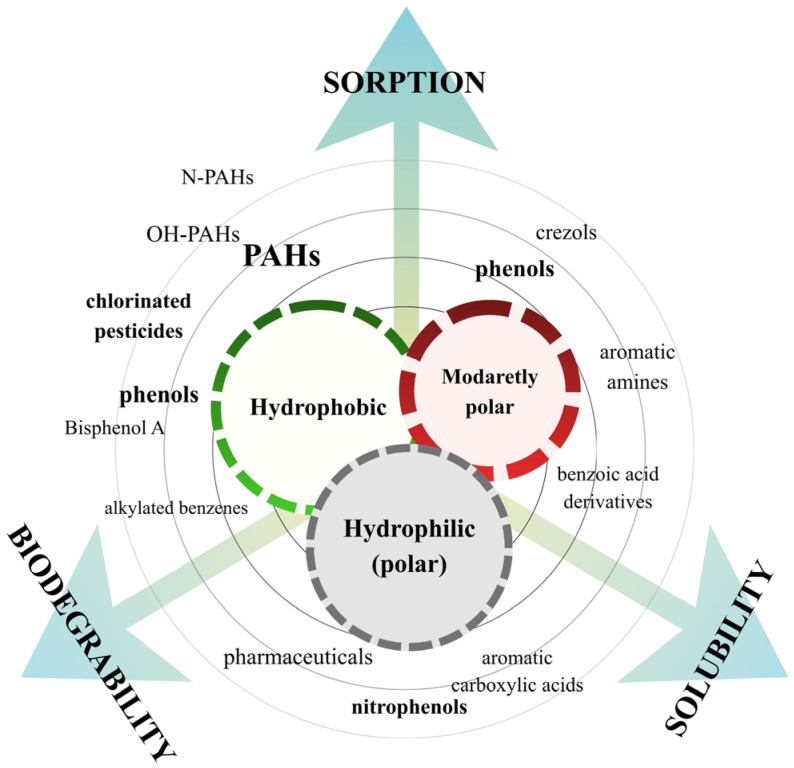
Classification of AOCs by physicochemical properties.

Aromatic organic contaminants (AOCs) are a subclass of environmental contaminants characterised by the presence of at least one aromatic ring in their molecular structure. Currently, the key AOCs identified in wastewater include polycyclic aromatic hydrocarbons (PAHs); their nitrated (N-PAHs) and hydroxylated (OH-PAHs) derivatives; phenolic compounds such as phenol, bisphenol A (BPA), and alkylphenols; and organochlorinated pesticides (OCPs), including Dichlorodiphenyltrichloroethane (DDT), Dichlorodiphenyldichloroethylene (DDE), chlordane, and lindane [16,17,18,19]. These groups originate from a wide range of anthropogenic activities: PAHs and their derivatives arise mainly from fuel combustion and industrial thermal processes; phenolic compounds are derived from plastic manufacturing, detergents, and consumer products; and OCPs reflect historical and ongoing agricultural applications [20,21]. Their presence in wastewater therefore represents the combined impact of industrial emissions, agricultural inputs, urban runoff, and domestic chemical use. The polarity of AOCs governs hydrophobicity and solubility, and thereby strongly influences affinity toward WW-MPs. On this basis, they can be grouped into three categories, which are depicted in Figure 2: hydrophobic (non-polar), moderately polar, and hydrophilic (polar) compounds.

Hydrophobic AOCs, including generally high-molecular-weight PAHs (HMW-PAHs) and OCPs, characteristically exhibit high octanol-water partition coefficients (log Kₒw) and considerably low aqueous solubility, which enhances the drive for sorption onto suspended WW-MPs [22]. However, adsorption prediction cannot rely solely on Kₒw, as compound-specific traits (size, charge, aromaticity), solution chemistry, and MP surface properties also strongly influence adsorption [23]. In general, less polar AOCs, including low-molecular-weight PAHs (LMW-PAHs), cresols, aromatic amines, and benzoic acid derivatives, represent lower values of log Kₒw and contain functional groups such as hydroxyl (-OH) or amino (-NH_2_) groups. Their increased water affinity enables interactions with WW-MPs mainly via relatively weak hydrogen bonding and π-π interactions [24]. Finally, hydrophilic AOCs, e.g., nitrophenols, aromatic carboxylic acids, and selected pharmaceuticals, are highly soluble, exhibit the lowest log Kₒw, and occur in the aqueous phase, often in ionised form, and among all AOCs, they show the weakest sorption to MPs [25]. Their environmental half-life parameter (t_1/2_), defined as the time required for 50% degradation, also varies substantially among compound classes. AOCs’ parameters and their corresponding physicochemical patterns are summarised in Table 1.

Highly hydrophobic AOCs preferentially partition into the sludge phase, resulting in notable accumulation that can appear as mass gains during treatment. Seasonal effects further modulate this behaviour: In summer, longer sludge retention and reduced biodegradation promote the build-up of LMW-PAHs and their hydroxy derivatives, such as naphthols [26]. In contrast, lower N-PAH concentrations in winter wastewaters indicate more efficient removal at low temperatures, consistent with the behaviour reported for HMW-PAHs [27]. Overall, only a limited portion of the total PAHs is removed via biodegradation, biotransformation, or volatilisation. Secondary formation processes, especially the conversion of parent PAHs to their metabolites, account for much of the observed mass increase [26,27]. Therefore, sewage sludge serves as a long-term storage matrix in which both hydrophobic AOCs and WW-MPs accumulate, increasing the potential for sustained sorption and joint environmental release.

**Table 1:** A comparative study of key physicochemical properties, removal, and biodegradability of major wastewater AOCs in average ranges.

AOC Class	log Kₒw	Water Solubility (25 °C), mg/L	pKa *	Removal Mechanism and Efficiency (2000–Present)	Reported Environmental Half-Lives	References
PAHs	3.3–6.7	<0.001–30	>25–40	sorption to activated sludge and dissolved organic matter (37–99%, Europe)	>3–180 d (surface water) 30–360 d (soils) 17–126 d (sewage sludge) 135 d–4.4 years (lake sediments)	[18,28,29,30,31]
OH-PAHs	2.7–5.63	0.09–866	7–10	sorption to activated sludge and dissolved organic matter (75–99%, Europe)	<5.2 d (surface water)	[32]
N-PAHs	2.3–5.74	0.003–88	~5–6 (basic), or non-ionisable	sorption to activated sludge and dissolved organic matter (37–83%, China)	4–60 d (surface water) 40–360 (soil) 200 d–3.5 years (lake sediments)	[27,31]
Phenols	1.46–4.21	<30,000 (substituted phenol derivatives),80,000 (unsubstituted phenols)	5–11	Biodegradation during activated sludge process or trickling filters (69–100%, Africa)	10–30 d (surface water) 1–2 d (soil)	[33,34,35]
OCPs	4.5–6.96	0.025–2000	non-ionisable	sorption to activated sludge and dissolved organic matter (37–100%, Europe)	1 d–30 years (surface water)	[36,37,38,39]

* pKa Data Compiled by R. Williams. ACS Division of Organic Chemistry, https://organicchemistrydata.org/hansreich/resources/pka/pka_data/pka-compilation-williams.pdf, updated 7 April 2022, assessed 25 November 2025.

A clear trend emerges: Substances with a high log Kₒw and low solubility, such as PAHs and some OCPs, are consistently removed from wastewater by sorption rather than microbial degradation, leading to their accumulation in sludge and potential for deposition on WW-MP surfaces. Moderately polar AOCs show mixed behaviour, with partial microbial transformation but persistent particulate-bound fractions. In contrast, the most soluble and polar phenols, as well as several PAH derivatives, remain mainly in the dissolved phase and are more susceptible to biodegradation, resulting in substantially higher removal efficiencies. The balance between hydrophobicity, solubility, and biodegradability determines the behaviour of selected AOCs in wastewater and explains the likelihood of partitioning onto WW-MP surfaces.

## 3. Wastewater-Derived Microplastics (WW-MPs): Transformation and Ageing

The wastewater stream serves as a significant transport pathway for MPs entering the natural environment. WW-MPs occur in various morphotypes, including fibres, fragments, films, spheres, and foams, and may be partially or entirely degraded during treatment [40]. In general, the most frequently reported polymer types in WWTPs globally include low- and high-density polyethylene (LDPE, HDPE), polypropylene (PP), polystyrene (PS), and polyethylene terephthalate (PET), with polycarbonate (PC) also detected in smaller amounts [41]. Their environmentally altered surfaces are shown in Table 2.

Reported particle sizes for WW-MPs range from a few micrometres to 5 mm, with colours including white, black, brown, transparent, and multicoloured [44,45]. The concentrations of WW-MPs in wastewater influents and effluents vary considerably, depending on factors such as the size of the served population, the characteristics of the catchment area, and the presence of point sources, including industrial discharges, which may contribute additional loads. Effluent MPs are typically smaller than influent particles due to both preferential removal of larger particles and in-plant fragmentation. Larger WW-MPs (>500 µm) are more efficiently removed, while smaller fractions (<250 µm, often <150 µm) dominate the final discharge as they can more easily flow through [46].

The latest studies by Nematollahi M.J. et al. (2025) and Alasvand S. et al. (2023) reported WW-MP levels in WWTP influent ranging from ~250 MPs/L in urban plants in Tabriz to over 700 MPs/L in Qom, Iran [47,48]. Okoffo et al. (2023) found higher particle concentrations in the influent of an Australian municipal WWTP, ranging from 840 to 3116 µg/L [49]. To illustrate the reduction in abundance after treatment, a study by Miserli K. et al. (2025) in Ioannina, Greece, reported 5.8 ± 0.6 MPs/L [42]. The reported mass concentration can range from about 0.71 to 1.75 µg/L in China and from 0.5 to 11.9 µg/L in Denmark [50,51]. Studies indicate an average efficiency of 90% in reducing WW-MPs from the main wastewater stream, which are retained in sewage sludge [15]. From here, depending on the treatment system (aerobic or anaerobic digestion), they may persist for up to 30 days before further management. In addition to heavy metals and other inorganic matter, sewage sludge also tends to accumulate AOCs, and during the relatively long stabilisation process, chemical and physical interactions with retained WW-MPs occur [52].

The WW-MP load composition discharged from WWTPs reflects both the dominant types of plastics in use and the specific point sources within the catchment area. Nevertheless, substantial local variations in the MP fingerprint of the effluent may occur, driven by differences in polymer composition, particle size, and specific morphology. Consequently, they exhibit diverse physicochemical properties that influence their mobility and interactions with co-occurring contaminants. In Greece, PA, polyacrylic acid (PAA), and a polyacrylamide-co-polyacrylic acid copolymer (PAM-co-PAA) were detected in 100% of sewage samples, reflecting a persistent local source. A high abundance of PVC, PE, PS, and PP was also detected there in 20–80% of samples [42]. Similarly, a study of a large WWTP in Northern Italy found that the effluent WW-MPs were predominantly polyesters (35%) and PA (17%) [53]. A global review by Yaseen A. et al. (2022) of 121 WWTPs found PE (22%), PS (21%), and PP (13%) to be the most frequently detected polymers in final effluents, with similar findings at national and regional scales [40]. Another review by Acarer A. et al. (2024) in Turkey also identified polyvinyl chloride (PVC) and polyamide (PA) as important polymers that could be detected easily in wastewater [54]. These studies confirm the occurrence of the most common microplastic polymers identified in recent EU reports [55].

Industrial streams generally carry significantly higher concentrations of WW-MPs; however, they exhibit limited characteristics specific to the process and waste generated [16,17,18]. Their effluents often have an elevated chemical oxygen demand (COD) and relatively low biochemical oxygen demand (BOD), with a lower COD/BOD ratio indicating more limited biodegradability and reflecting the presence of organic compounds resistant to degradation, including WW-MPs [56,57]. Case studies comparing different wastewater streams provide clear evidence: In Cádiz, Spain, effluent from a municipal WWTP contained 16.4 MPs/L, whereas effluent from a nearby industrial WWTP contained 131.35 MPs/L, an eight-fold increase [58]. A study in Xiamen, China, showed that industrial wastewater carried more than two times the MP load of domestic sewage, resulting in a 3.2-fold increase in the annual discharge flux [59]. A study from Vietnam confirmed this pattern, with industrial wastewater containing ~60.9 MPs/L, compared with ~31.5 MPs/L in domestic streams and ~35.5 MPs/L in medical wastewater, respectively [60]. A recent meta-analysis further supports the conclusion that WWTPs receiving substantial industrial inputs exhibit influent WW-MPs concentrations that are significantly higher than those of municipal systems alone [61].

Evaluation of the industrial process and quantitative characterisation enable approximate predictions of WW-MP loads, allowing adjustments to the treatment process to minimise emissions. However, exact estimates are not possible for WW-MPs of municipal origin due to the specific location of WWTPs, greater variability in wastewater chemical composition, and, notably, the direct unsuitability of municipal WWTPs for targeted removal of this type of solid matter. Beyond abundance, industrial WW-MPs also differ in morphology, polymer composition, and predictable surface condition, all of which are critical determinants of resultant condition and sorption affinity for AOCs. In Xiamen, MPs from industrial effluents were predominantly fibrous with markedly rougher surface textures, whereas MPs from domestic wastewater were mainly granular [59]. Industrial streams were enriched in polyester (PES) and PET particles-polymers associated with textile and manufacturing activities. This source-specific enrichment is supported by findings from Guangzhou, where the number of textile factories in a WWTP catchment was positively correlated with total MP release [62]. Fibrous MPs typically present larger surface-area-to-volume ratios, more oxygen-containing functional groups after oxidative ageing, and higher affinity for biofilm colonisation than more granular domestic MPs [63,64]. As a result, industrial WW-MPs are more likely to develop heterogeneous, chemically active surfaces that bind AOCs more effectively. Control over the general MP cycle is further complicated by the use of treated sewage sludge (biosolids) as fertilisers, whose MP loads are often unknown, and which re-enter the environment with associated co-micropollutant loads [65,66].

### 3.1. Morphological Alterations

WW-MP size reduction, altered surface roughness, and partial fragmentation are commonly due to the high shear stress from turbulence induced by continuous mixing or aeration during treatment. This generates forces that mechanically abrade particles’ surfaces, reducing their total mass and increasing the abundance of smaller WW-MP fractions [67]. Gravity-based separation processes, such as primary sedimentation, preferentially remove denser, more compact WW-MP shapes, such as spheres and fragments, which readily settle into sludge [68]. High-density polymers, including PVC (d = 1.30–1.45 g cm^−3^) and PET (d = 1.33–1.38 g cm^−3^), go through a similar process [69,70]. In contrast, fibres and low-density polymers, such as PE (d = 0.91–0.96 g cm^−3^) and PP (d = 0.83–0.85 g cm^−3^), with their high aspect ratio and generally low density, tend to remain suspended, resulting in their high proportion in treated effluents [71,72]. As a result, final effluents are typically enriched in fibrous, low-density plastics, though some biofilm-covered WW-MPs may also escape removal [73]. Therefore, evaluating WW-MP removal efficiency should consider both mass concentration and particle size distribution.

### 3.2. Chemical Alterations

During treatment, WW-MPs are subjected to conditions that promote measurable physicochemical changes relevant to their sorption behaviour. One of the most critical operational parameters is hydraulic retention time (HRT), which allows extended contact between WW-MPs and AOCs. HRT typically ranges from several hours to about 1 day, during which surface abrasion and microcrack formation extend, thereby increasing the number of accessible sorption sites to contaminants. The typical WWTP operational pH range of 6.5–8.5 facilitates the hydrolysis of condensation polymers such as PET, leading to chain scission and the formation of -COOH- and -OH-containing end groups [74]. These modifications alter surface polarity and can enhance interactions with AOCs [75]. Similarly, exposure to aerobic conditions (up to 2 mg/L in typical WWTP) promotes oxidative modification of WW-MPs surfaces, whereas reduced oxygen zones favour the onset of bacterial deposition [76]. WW-MPs are further exposed to coagulants, surfactants, and more oxidants (e.g., chlorine, ozone), which can further alter surface charge, facilitating subsequent sorption of AOCs [77,78].

The increase in reactivity of damaged (aged) MP surfaces was demonstrated by Fernando S. et al. (2007) for polyolefin PE, PP, and PS fragments, which frequently acquire oxidised aromatic structures that strengthen interactions with AOCs [79]. The oxidative ageing of polymer surfaces was thoroughly analysed by Duan J. et al. (2022), who reported that reactive oxygen species (ROS), including hydroxyl radicals (•OH), singlet oxygen (^1^O_2_), and superoxide (O_2_•^−^), generated during chlorination, ozonation, and microbial treatment, are key drivers of polymers’ transformations in WWTPs [72]. ROS “attack” polymer backbones and side groups, leading to chain scission and the formation of oxygen-containing functional groups. In polyolefins, this results in increased surface polarity and roughness, while in aromatic polymers, it promotes aromatic ring oxidation and the formation of quinone-like structures. For condensation polymers, ROS accelerate the cleavage of ester and carbonate bonds, thereby enhancing hydrolysis [72]. As a result, the sorption behaviour of WW-MPs toward AOCs shifts as the affinity for highly hydrophobic compounds tends to decrease, whereas moderately polar aromatic compounds can exhibit enhanced sorption due to the formation of specific polar and aromatic interactions. Table 3 summarises chemical transformations of common WW-MPs that can occur during wastewater treatment and their potential effects on sorption properties toward AOCs. These wastewater-induced modifications generally reduce polymers’ hydrophobicity, mechanically degrade particles, creating cracks and pores, and broaden MPs’ sorption capacity by increasing their specific surface area [80].

**Table 3:** Reported wastewater-induced chemical transformations of common polymers, excluding biofilm effect.

Polymer Group		MP Polymer Type	Dominant Bonding Mechanism	Typical Transformation During Wastewater Treatment	Resulting Changes	General Sorption Effect	Sorption Effect Toward AOCs	References
Addition polymers (non- or weakly polar)	Vinyl-aromatic	PS-WW-MPs	Aromatic π-π interactions, C-C backbone structure	Oxidation (ROS), UV ageing, aromatic ring oxidation, chain scission, chlorination/sulfonation (minor)	Developing oxidised aromatic structures (quinones, hydroxylated rings)	↑ Polarity↑ Hydrophilicity= enhanced π-π interactions (PS) = enhanced H-bonds and dipole–dipole interactions (PE, PP)	↑ Affinity for hydrophobic compounds (e.g., PAHs) ↑ Affinity for moderately polar organics (e.g., BPA, phenols)	[72,81,82,83]
	Polyolefins	PE-WW-MPs	C-C backbone, weak van der Waals	Oxidation (ROS), UV/photo-oxidation, biofilm colonisation, oxidation, chain scission, surface cracking	Developing of carbonyl (C=O), hydroxyl (-OH), carboxyl (-COOH) surface groups; surface cracking, pitting			[84,85,86]
		PP-WW-MPs	C-C backbone with pendant -CH_3_ groups	Oxidation (aerobic/anoxic cycling), hydroperoxide formation, surface embrittlement	Developing hydroxyl, fewer carbonyl structures, and chain scission			[83,87]
Condensation (polar)		PET-WW-MPs	Ester linkages (-COO-), polar	Hydrolysis (pH 6.5–8.5), oxidation	Developing hydroxyl and carboxyl end groups from ester cleavage; increased polarity	↑ Polarity↓ Hydrophobicity = enhanced π-π interactions	↓ Affinity for strongly hydrophobic compounds (e.g., PAHs) ↑ Affinity for moderately polar organics (e.g., BPA, phenols)	[88,89,90]
		PC-WW-MPs	Carbonate ester linkages (-O-CO-O-)	Hydrolysis of carbonate linkages, UV/photo-oxidation	Development of hydroxyl and carboxyl groups; chain cleavage			[91,92,93]

## 4. Environmental Matrix Effects on AOC Sorption by WW-MPs

AOCs associate with MPs primarily through hydrophobic interactions, van der Waals forces, and π-π stacking between aromatic structures. Depending on the surface chemistry of the particles, electrostatic interactions and hydrogen bonding may also contribute, and for certain OCPs, halogen bonding has additionally been reported [94]. A general trend for pristine MPs reported widely in the literature is that sorption increases with decreasing particle size (and thus increasing specific surface area), and that weathered MPs exhibit higher sorption capacities. However, in wastewater systems, these relationships are often non-linear, as they are strongly modulated by matrix-related environmental factors. Variations in pH, ionic strength/salinity and temperature, and the presence and character of dissolved organic matter (DOM), as well as biofilm development on WW-MP surfaces, can modify MP surface charge, the abundance and type of functional groups, and diffusion processes, thereby influencing overall sorption efficiency [95,96]. These environmental factors affect not only AOC behaviour (solubility, speciation, partitioning) but also WW-MP surface properties, and thus jointly control the efficiency and selectivity of AOC-WW-MP complexation [88,95]. By regulating the charge state of functional groups, altering contaminant solubility and ionisation, and introducing competitive and non-equilibrium interactions with other sorbates (e.g., DOM, co-contaminants), they ultimately govern the distribution of AOCs between the aqueous phase and WW-MP surfaces. To ensure relevance for micropollutant transport, comparisons of sorption capacity should therefore be based on environmentally or at least artificially aged MPs in aqueous systems, rather than on untreated, pristine materials. Substantial variability within these groups is expected due to differences in degradation history prior to entering the wastewater stream and residence time within the treatment system. Nevertheless, such a classification enables approximate estimations and comparative assessments of AOC sorption behaviour within a given environmental domain.

### 4.1. Effects of pH and Ionic Strength

pH is one of the most influential factors governing AOC-WW-MP interactions because it simultaneously controls the surface charge of the polymer and the ionisation state of pH-sensitive contaminants. Sun et al. (2022) [97] demonstrated that acidic conditions enhance the adsorption of ionisable pharmaceuticals onto MPs, as protonation reduces electrostatic repulsion and promotes hydrophobic and hydrogen bonding interactions. In municipal wastewater, which typically exhibits a near-neutral pH, oxygen-containing functional groups formed during MP ageing are largely uncharged. In contrast, biofilm-coated WW-MPs acquire a predominantly negative charge due to the extracellular polymeric substances (EPSs) associated with microbial colonisation [98]. Non-ionisable hydrophobic PAHs are largely unaffected by pH; however, phenols and hydroxylated PAHs exhibit pronounced pH-dependent behaviour driven by their pKa values. Below their pKa, these compounds remain neutral and hydrophobic, favouring adsorption to MPs. Above their pKa, they become anionic and more water-soluble, reducing their affinity for MP surfaces [27,99]. At elevated pH, the combination of negatively charged (biofouled or oxidised) MP surfaces and deprotonated AOCs results in electrostatic repulsion and diminished sorption [100]. Conversely, under neutral to mildly acidic conditions, typical for domestic wastewater, electrostatic barriers are weaker, enabling sorption through hydrophobic interactions, π-π stacking, and hydrogen bonding.

Ionic strength is the second major physicochemical factor influencing AOC sorption onto WW-MPs. Increasing ionic strength reduces the thickness of the electrical double layer, thereby diminishing electrostatic repulsion between charged MP surfaces and ionisable AOCs [101]. This screening effect facilitates the approach of anionic contaminants to negatively charged aged or biofilm-coated WW-MPs, enabling sorption pathways that would otherwise be inhibited under low-ionic-strength conditions. At the same time, common wastewater cations such as Na^+^, Ca^2+^, and Mg^2+^ interact with MP surfaces in multiple ways. Monovalent ions primarily screen surface charge, whereas divalent cations can form inner-sphere complexes, bridge functional groups, or compete with cationic AOCs for negatively charged sorption sites [102]. Such competitive and site-blocking processes may reduce the net sorption of positively charged or protonated contaminants. For non-ionisable hydrophobic AOCs (e.g., PAHs), higher ionic strength increases sorption by decreasing their activity in water and enhancing hydrophobic partitioning. However, because wastewater ionic strength is moderate (typically 0.01–0.1 M), the overall effect tends to arise from a balance between hydrophobic enhancement, electrostatic screening, and cation competition. Consequently, ionic strength-driven sorption responses are often non-linear and contaminant-specific rather than uniformly promotive or inhibitory [103,104].

### 4.2. Effect of Dissolved Organic Matter (DOM)

Humic and fulvic substances (e.g., humic acid (HA) and fulvic acid (FA)) represent a third major factor modulating AOC sorption in wastewater systems [105]. Owing to its heterogeneous composition, DOM contains both hydrophobic aromatic domains and hydrophilic domains with highly functionalised regions, allowing interactions with a broad spectrum of AOCs. Hydrophobic contaminants may partition into the non-polar domains of DOM colloids, whereas more polar or ionisable AOCs interact through hydrogen bonding, π-π interactions, or electrostatic attraction with DOM functional groups. These processes reduce the freely dissolved contaminant fraction and thereby limit its direct sorption onto WW-MP surfaces [106,107,108]. Simultaneously, DOM can adsorb onto WW-MPs, forming a dynamic organic coating analogous to early biofilm conditioning layers. Such coatings can introduce additional oxygenated functional groups and physically block sorption sites, collectively reducing the accessibility of the underlying polymer surface [102,103]. Although DOM typically suppresses direct AOC sorption onto WW-MPs, it may enhance the overall retention of contaminants through the formation of mobile WW-MP-DOM-AOC complexes, which remain suspended within the wastewater matrix. Thus, the influence of DOM is dual: it decreases surface-bound sorption yet can increase contaminant association with MPs via indirect complexation pathways.

### 4.3. Effects of Biofilm Formation and Temperature

Microbial activity represents another critical factor shaping AOC-WW-MP interactions, particularly in activated sludge systems. Here, high microorganism concentrations promote rapid biofilm formation on particle surfaces. Biofilms are structured microbial consortia embedded within a self-produced EPS matrix, enhancing cell adhesion and protecting against environmental stressors [109]. The resulting biofilm-coated MPs, often referred to as the plastisphere or ecocorona [110], constitute a highly functionalised secondary sorbent phase. EPSs introduce abundant -COOH, -OH, and -NH_2_ groups derived from proteins, polysaccharides, and humic substances, which confer a predominantly negative surface charge under typical wastewater pH (6–8) conditions [73]. Biofilm coatings significantly modify sorption behaviour. Bhagat et al. (2024) demonstrated that PP/PE MPs colonised for 15 days sorbed substantially more phenanthrol (0.07 mg/g) compared with pristine MPs (0.001 mg/g), whereas phenanthrene sorption remained unaffected, indicating a contaminant-specific biofilm influence [111]. Similarly, Cui et al. (2023) reported that biofouled HDPE MPs exhibited a pronounced, multi-fold increase in sorptive capacity relative to pristine particles, highlighting the complex interplay between chemical sorption and biofilm-mediated processes [23]. Beyond altering surface chemistry, biofilms create hotspots for horizontal gene transfer, facilitating the exchange of genetic material, including antibiotic resistance genes (ARGs), and thereby contributing to the spread of antimicrobial resistance [112,113]. The extent of biofilm development varies among polymers. Kwiatkowska and Ormaniec [114] showed that PP-MPs supported the most stable biofilm formation, whereas PE-MPs exhibited greater affinity for coliform bacteria. Despite polymer-specific differences, biofouled WW-MPs exhibit an enhanced capacity to bind, accumulate, and transport AOCs within wastewater. This vector effect effectively transforms WW-MPs into “Trojan horses” capable of delivering concentrated contaminant loads into receiving environments, increasing both bioavailability and toxicological risk for exposed organisms [73,100,115,116,117].

Research on the influence of wastewater temperature on AOC-WW-MPs is still limited in the available literature. Although temperature is expected to play an important role, it remains overlooked in experimental studies. Biofilms respond strongly to thermal conditions: elevated temperatures stimulate microbial metabolism, EPS formation, and enzymatic activity, generating surfaces with a higher density of reactive functional groups that can enhance the sorption of polar or ionisable contaminants [111]. In real wastewater systems, temperatures typically range between 10 (winter) and 25 °C (summer), depending on treatment stage, and these fluctuations can influence both sorption kinetics and thermodynamics. However, empirical data remain scarce. A study by García-Pimentel et al. (2022) [118] reported no significant temperature effect on the sorption of m-CPF to HDPE-MPs, indicating that thermal sensitivity may be compound-specific and therefore relatively weak for certain pesticides.

**Figure 3:**
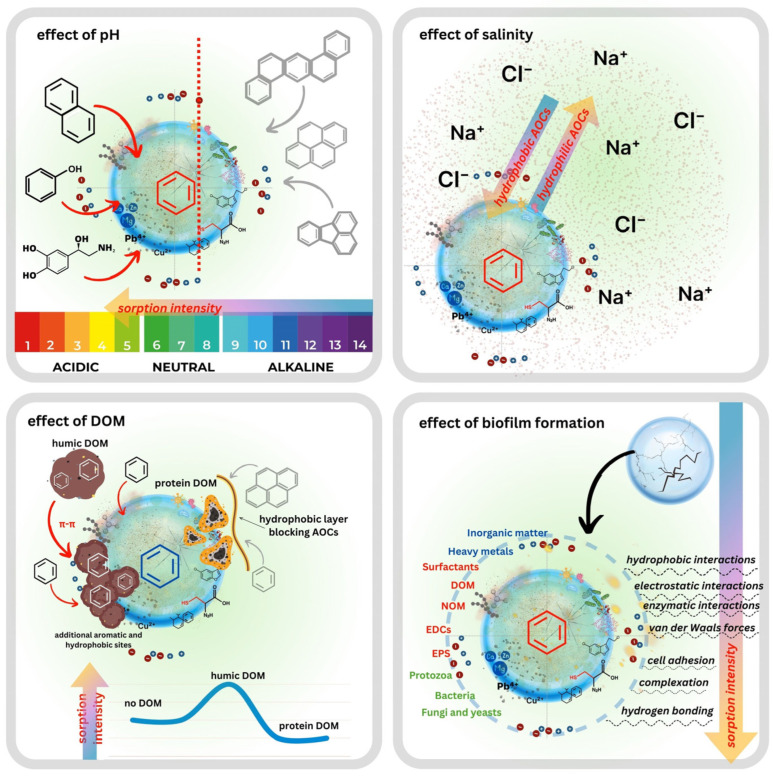
Environmental factors in wastewater influencing AOC-WW-MP interactions.

The above-described environmental factors are illustrated in Figure 3.

## 5. Sorption Models for WW-MP-AOC Systems

### 5.1. Available Modelling Approaches

Assessing sorption behaviour under wastewater conditions remains difficult, as WW-MPs exhibit substantial chemical and structural variability depending on effluent composition and treatment methods. Their diverse heterogeneous properties and complex solution chemistry, together with the lack of standardised MPs release limits, have restricted the development of modelling frameworks explicitly tailored to wastewater systems. As a result, most sorption models applied to WW-MPs are derived from simplified aqueous environments that can be reproduced in laboratory conditions. Among them, classical equilibrium and kinetic approaches remain widely used. Equilibrium isotherms, including the Freundlich, Langmuir, Temkin, and Dubinin-Radushkevich models, are applied to characterise sorption capacity and the heterogeneity of binding sites. At the same time, kinetic models such as the Elovich, Intraparticle Diffusion, Film Diffusion, Pseudo-First-Order, or Pseudo-Second-Order approach describe the rate-limiting steps and overall uptake dynamics. Combined models, such as the Langmuir-Freundlich (dual-mode) description, have been used for aged or biofilm-coated MPs to account for the coexistence of surface adsorption and polymer-phase partitioning, and the Redlich-Peterson and Dubinin-Radushkevich models may provide better fits for structurally altered particles.

### 5.2. Limitations of Laboratory-Derived Sorption Models

Despite several developed MP-AOC modelling approaches, the assumptions underlying these models, such as homogeneous surfaces, single-solute systems, and near-equilibrium conditions, are rarely met in wastewater matrices. An analysis of the laboratory-derived isotherms from Table 4 showed that experiments are typically generated under controlled, single-solute conditions that do not cover the complexity of wastewater composition, where multi-solute competition and dynamic redox and hydraulic regimes strongly influence sorption processes. In real systems, AOCs coexist with surfactants, humic substances, pharmaceuticals, metals, and colloids that compete for the same binding sites, suppressing or altering sorption behaviours that appear favourable in simplified laboratory settings. Moreover, laboratory studies on sorption models typically assume direct equilibrium conditions, whereas wastewater rarely presents a stable, steady state. Continuous flow with daily and hourly fluctuating contaminant loading and temporal variability in suspended solids drive sorption processes governed by non-equilibrium kinetics, boundary-layer resistance, and slow intra-polymer diffusion. These mechanisms are not accountable by conventional isotherms. Consequently, sorption capacities can be overestimated and underestimate many competitive interactions, as well as resulting parameter values that are not directly applicable to operational wastewater environments. Current modelling efforts therefore rely largely on single-AOC, single-MP systems in controlled media conditions, where heterogeneity is represented mathematically and the combined effects of ageing, biofilm development, and complex wastewater chemistry cannot be reproduced. Some degree of simplification is indeed necessary to any modelling approach; however, these limitations highlight the need for frameworks calibrated under more realistic environmental conditions that more accurately reflect wastewater treatment system phenomena. A robust evaluation of AOC-WW-MP interactions thus requires integrating equilibrium and kinetic modelling with a detailed physicochemical characterisation of both the MPs and the surrounding wastewater matrix, including polymer ageing, multi-solute competition, and variable solution chemistry.

Therefore, wastewater-relevant model adaptations should undertake to incorporate the following four key factors simultaneously:

(i) Ageing-induced MP surface heterogeneity;
(ii) Competitive sorption of co-contaminants onto MPs driven by specific medium chemistry;
(iii) Non-equilibrium AOC sorption behaviour associated with redox system variability;
(iv) Altered diffusion behaviour within weathered MPs.

Based on the identified parameters i–iv, it seems like the dual-mode Langmuir-Freundlich formulation comes closest to capturing features relevant to WW-MPs, as it integrates heterogeneous surface sorption with a partitioning term that accounts for polymer-phase diffusion. Nevertheless, this model still lacks terms for competitive, non-equilibrium sorption with dissolved organic matter or co-occurring pollutants and does not capture the substantial hydraulic and redox variability present in wastewater treatment systems. As such, all existing models provide only partial representations of real WW-MP-AOC interactions and must be interpreted within the constraints of their underlying assumptions.

Table 4 summarises the principal isotherm and kinetic models used to describe sorption of MPs-AOCs, which are, at present, the only available formulations applicable to WW-MP-AOC systems. It indicates each model’s domain of applicability and clearly identifies which crucial wastewater-relevant factors (i–iv) each model fulfils.

**Table 4:** Overview of equilibrium, kinetic, and hybrid modelling frameworks used to characterise AOC-MP systems, including their governing equations, key parameters, mechanistic interpretations, and representative studies (2020-2025).

Model	Descriptive Equation	Wastewater-Relevant Factors Incorporated (i-iv)	Specifics for WW-MP-AOC Sorption	Relative Popularity	References
Freundlich (equilibrium)	$qₑ\;=\;K_{F}\;C{ₑ}^{\frac{1}{n}}$ where $q_{e}$-adsorbed amount at equilibrium (mg/g) $C_{e}$-equilibrium concentration (mg/L) $K_{F}$-Freundlich constant (adsorption capacity) n-heterogeneity factor (1/*n* < 1 indicates favourable adsorption)	(i)	Captures heterogeneous sorption arising from oxidised and roughened WW-MP surfaces	Very high-most widely applied to PAHs and phenols	[119,120,121]
Langmuir (equilibrium)	$qₑ\;=\frac{\left(q_{\max\;}\;K_{L}\;Cₑ\right)}{\;}\left(1\;+\;K_{L}Cₑ\right)$ where $q_{e}$-adsorbed amount at equilibrium (mg/g) $q_{max}$-maximum adsorption capacity (mg/g) $K_{L}$-Langmuir constant related to binding energy (L/mg) $C_{e}$-equilibrium concentration (mg/L)	none	Assumes ideal monolayer sorption, does not reflect AOC-WW-MP systems	High-often used alongside the Freundlich model for model comparison	[121,122]
Temkin (equilibrium)	$qₑ\;=\;B\ln\left(ACₑ\right)$ where $q_{e}$-adsorbed amount at equilibrium (mg/g) A-Temkin equilibrium binding constant (L/g) $B=\frac{RT}{b}$-constant related to heat of adsorption (J/mol) R-universal gas constant (8.314 J/mol K) T-temperature (K) b-Temkin constant related to adsorption energy (J/mol) $C_{e}$-equilibrium concentration (mg/L)	(i)	Represents decreasing adsorption energies consistent with chemically modified WW-MP surfaces	Moderate-mainly used in thermodynamic assessments	[123,124]
Redlich -Peterson (R-P) (equilibrium)	$qₑ\;=\frac{\left(K_{R}\;Cₑ\right)}{1\;+\;a_{R}\;C{ₑ}^{\beta }}$ where $q_{e}$-adsorbed amount at equilibrium (mg/g) $K_{R}$-R-P constant (L/g) $a_{R}$-adsorption energy constant (L/mg) β-exponent (0 < β < 1), indicating hybrid Langmuir-Freundlich behaviour $C_{e}$-equilibrium concentration (mg/L)	(i)	Hybrid behaviour suitable for partially aged or heterogeneously modified WW-MP surfaces	Moderate-fits PAH, BPA, and phenolic compound sorption more accurately than single two-parameter models	[123]
Dubinin-Radushkevich (D-R) (equilibrium)	$qₑ\;=\;q_{m}\exp\left(-B\varepsilon^{2}\right)$ $\varepsilon \;=\;RT\ln\left(1\;+\frac{1}{Cₑ}\right)$ where $q_{e}$-adsorbed amount at equilibrium (mg/g) $q_{m}$-theoretical monolayer capacity (mg/g) B-D-R constant related to adsorption energy (mol^2^/J^2^) ε-Polanyi potential (J/mol) $E=(2B{)}^{-1/2}$-mean free energy of adsorption (kJ/mol) $C_{e}$-equilibrium concentration (mg/L)	(i)	Describes energetically non-uniform sorption typical of structurally altered WW-MPs	Moderate-supplementary model in PAH sorption studies	[120,125]
Pseudo-First-Order (PFO) (kinetic)	$\ln\left(qₑ\;-\;q_{t}\right)=\ln qₑ-\;k_{1}t$ where $q_{e}$-adsorbed amount at equilibrium (mg/g) $q_{t}\;$-adsorbed amount at time t (mg/g) $k_{1}$-rate constant of first-order adsorption (1/min) t-contact time (min)	(iii)	Represents early uptake stages of hydrophobic AOCs; suitable for smooth polymer surfaces	High-used as baseline in kinetic fitting to describe early uptake stages of hydrophobic AOCs	[126]
Pseudo-Second-Order (PSO) (kinetic)	$\frac{t}{q_{t}}=\frac{1}{k^{2}q{ₑ}^{2}}+\frac{t}{qₑ}$ where $q_{e}$-adsorbed amount at equilibrium (mg/g) $q_{t}$-adsorbed amount at time t (mg/g) $k_{2}$-rate constant of second-order adsorption (g/mg min) t-contact time (min)	(iii)	Represents time-dependent sorption dominated by non-equilibrium surface processes on WW-MPs	Very high-most widely reported kinetic model with best fit for PAHs and phenols	[127]
Elovich (kinetic)	$q_{t}=\left(\frac{1}{\beta }\right)\ln\left(\alpha \beta \right)+\left(\frac{1}{\beta }\right)\ln\left(t\right)$ where $q_{t}$-adsorbed amount at time t (mg/g) α-initial adsorption rate (mg/g min) β-desorption constant related to surface coverage (g/mg) t-contact time (min)	(iii)	Describes non-equilibrium surface-controlled sorption typical of aged, chemically diverse WW-MPs	Moderate-used in advanced kinetic modelling	[125]
Intraparticle Diffusion (Weber-Morris, W-B) (kinetic)	$q_{t}=\;k_{id}\;t^{0.5}+\;C$ where $q_{t}$-adsorbed amount at time t (mg/g) t-contact time (min) -intraparticle diffusion rate constant (mg/g min^2^) C-boundary layer thickness (mg/g)	(iii) (iv)	Captures diffusion-controlled uptake influenced by microcracks and porosity in weathered WW-MPs	High-typically used as a diagnostic tool after PSO fitting; good fit to PAHs and aged MP-PS/PVC	[128]
Film Diffusion (kinetic)	$\ln\left(1-F\right)=\;-k_{f}t$ where $F=\frac{q_{t}}{q_{e}}$-fractional adsorption at time t $k_{f}$-film diffusion rate constant (1/min) t-contact time (min)	(iii)	Represents boundary-layer limitations relevant under fluctuating hydraulic conditions in WWTPs	Moderate-used to verify external diffusion contribution	[129]
Dual-Mode (Langmuir-Freundlich, equilibrium)	$qₑ\;=\frac{q_{s}\left(bCₑ\right)}{1\;+\;bCₑ}+\;K_{F}C{ₑ}^{\frac{1}{n}}$ where $q_{s}$-saturation capacity of Langmuir-type sites (mg/g) b-Langmuir affinity constant (L/mg) $K_{F}$-Freundlich constant (adsorption capacity) n-heterogeneity factor (1/*n* < 1 indicates favourable adsorption)	(i) (iv)	Combines heterogeneous surface sorption with polymer-phase uptake relevant for weathered WW-MPs	Moderate	[130]

### 5.3. Modelling Sorption Behaviour of Weathered MPs Under Wastewater Environmental Factors

Among the available modelling approaches, none simultaneously incorporates all four wastewater-relevant factors identified in this review. Nonetheless, several studies employing PSO kinetics have begun to include selected environmental parameters, offering partial insight into weathered MP behaviour under realistic conditions. Modelling approaches describing the sorption behaviour of weathered MPs under wastewater environmental factors are summarised in Table 5.

## 6. AOC-WW-MP Sorption, Ecological Exposure, and Risk: Key Implications and Research Gaps

Understanding how weathered MPs sorb AOCs under wastewater conditions is essential for evaluating their role as chemical carriers, yet such information is still rarely incorporated into ecological exposure or risk assessment frameworks. Sorption influences both the contaminant load bound to MPs and the environmental compartments or organisms to which these particles may subsequently be transported. Weathered MPs-typically more oxidised, biofilm-covered, and with altered surface charge-tend to bind AOCs more strongly, but the ecological consequences of this enhanced sorption remain poorly quantified. The chemical composition of MPs also affects their behaviour. Different base polymers and the presence of additives (e.g., plasticisers, stabilisers, antioxidants, surfactants, biocides) can modify sorption capacity and may themselves leach under changing pH, ionic strength, or temperature. PVC is a notable example: it contains high levels of DEHP, and its release under wastewater-like conditions has been experimentally demonstrated. Such additive leaching introduces additional organic contaminants into the system. Particle size further plays a role. Feng et al. (2020) showed that BPA and t-butylphenol leached more rapidly as epoxy MPs decreased from 1 mm to 100 nm due to a higher surface-to-volume ratio and greater density of sorption sites [135]. This is relevant in WWTPs, where smaller MPs and fragments are frequently detected and may therefore contribute disproportionately to contaminant transport. After leaving wastewater treatment, MPs bearing sorbed AOCs may enter receiving waters, sediments, or soils through effluent discharge or sludge application. These pathways align with established ecological exposure routes, including ingestion, trophic transfer, and sediment-water interactions. Sorption determines both the bioavailable fraction of contaminants in the surrounding medium and the chemical load delivered to organisms ingesting MPs. Weathering features-such as increased surface area, higher functional group density, and biofilm colonisation-can also enhance desorption in digestive fluids, increasing internal exposure beyond what water concentrations alone would predict. Despite these mechanistic insights, most current risk assessment frameworks evaluate AOCs independently of MPs. Few studies quantify how MP-associated transport alters predicted exposure concentrations or fate-and-transport behaviour. Likewise, ecological risk assessments seldom consider co-exposure, mixture toxicity, or modified bioaccumulation linked to MP-AOC complexes. Existing evidence suggests that MPs often act as short-term or episodic carriers rather than long-term sinks, yet this behaviour is not reflected in standard assessment approaches.

WW-MPs undergo extensive physicochemical transformation during treatment, generating sorption domains that differ fundamentally from those of pristine polymers. These changes directly influence the binding, retention, and mobility of AOCs in receiving environments, shaping exposure pathways and long-term environmental risk. However, research on truly environmental WW-MPs remains limited, and data on the long-term fate, bioavailability, and transformation of MP-AOC complexes remain scarce. The magnitude, variability, and system-specific character of these transformations are still poorly constrained across different WWTP configurations. Although ageing and biofilm development on WW-MPs have been widely documented, their integration into sorption modelling remains incomplete. Current isotherm and kinetic models typically incorporate only one or two of the four critical descriptors identified in this review-surface functionality, competitive sorption from co-contaminants, non-equilibrium interactions, and altered diffusion behaviour. As a result, predictions do not fully represent real wastewater conditions. Given the inherent variability of wastewater composition and the diversity of MP transformation processes, these factors must be explicitly incorporated; otherwise, mechanistic models cannot evolve toward greater environmental realism, limiting assessments of exposure or contaminant transfer relevant to wastewater-based epidemiology. Methodological inconsistencies also hinder progress. Comparative studies of WW-derived, field-aged, and laboratory-aged MPs reveal substantial discrepancies arising from differences in sampling strategies, isolation and separation procedures, ageing protocols, pre-treatment steps, and aqueous-phase chemistry. Without harmonised and validated workflows, cross-study comparisons remain largely interpretative, and model calibration remains unreliable. Standardising sampling, separation, surface characterisation methods, and test solution chemistry-particularly pH, ionic strength, DOM, and co-contaminant composition-is essential for generating comparable data.

A further priority is incorporating experimentally constrained sorption and kinetic parameters into fate-and-transport models. Research on PAHs and other hydrophobic AOCs demonstrates that including MP-associated sorption can substantially alter predicted residence times, phase partitioning, and downstream transport. Extending these modelling frameworks to represent the actual ageing state, porosity, and biofilm coverage of WW-MPs will enable more realistic assessments of AOC persistence and mobility in wastewater environments.

## Figures and Tables

**Table 2:** Examples of aged surfaces of common WW-MPs found in the wastewater or sewage sludge. Adapted from Refs. [42,43] under the terms of the Creative Commons Attribution (CC BY 4.0) Licence (https://creativecommons.org/licenses/by/4.0/).

WW-MP Polymer Type	Chemical Structure	Surface (SEM Image)	Reference
Polyethylene (PE-WW-MPs)	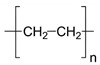	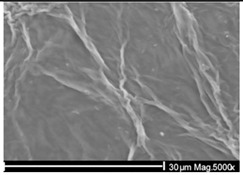 (extracted from wastewater)	[42]
Polypropylene (PP-WW-MPs)	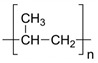	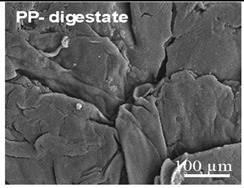 (extracted from sewage sludge)	[43]
Polystyrene (PS-WW-MP)	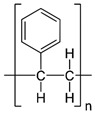	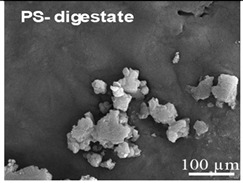 (extracted from sewage sludge)	[43]
Polyethylene terephthalate (PET-WW-MP)	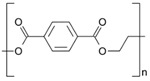	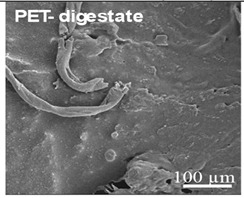 (extracted from sewage sludge)	[43]
Polyvinyl chloride (PVC-WW-MP)	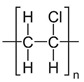	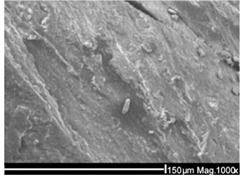 (extracted from wastewater)	[42]
Polyamide (PA-WW-MP)	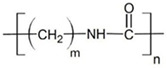	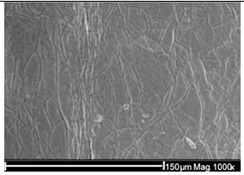 (extracted from wastewater)	[42]

**Table 5:** Overview of the sorption behaviour of weathered MPs considering environmentally relevant factors and unit-standardised metrics.

Study	MP Type and Size	Weathering Type	AOCs Tested	Equilibrium Time	pH Range and Effect	Ionic Strength/Salinity Range and Effect	Temperature Effect	DOM Effect	General Ageing Effect	Best-Fit Model and Parameters (Weathered MPs)
Liang et al., 2023 [131]	PS-MPs, PBAT-MPs (75–150 µm)	High-temperature (70 °C) + UV (15 d)	Diclofenac (DCF)	24 h	pH 3–9; aged MPs showed higher sorption across all pH. Sorption decreases as pH increases	0.6 M NaCl decreased sorption by 48.8% (A-PS) and 83.1% (A-PBAT). High salinity suppresses sorption via charge screening.	Not studied (room T)	35 mg/L HA reduced sorption by 24.3% (A-PS) and 20.4% (A-PBAT). DOM blocks active sites	Ageing increased DCF sorption 2–3×.	Freundlich: PS-MPs-DCF: K_F_ = 2.136 ± 0.402; n = 36.17 PBAT-MPs-DCF: K_F_ = 4.299 ± 0.343; n = 31.59 PSO: PS-MPs-DCF: k_2_ = 1.781 g mg^−1^ h^−1^; qₑ = 22.63 mg/g PBAT-MPs-DCF: k_2_ = 0.496 g mg^−1^ h^−1^; qₑ = 29.66 mg/g
Liu et al., 2019 [132]	PS-MPs, PVC-MPs (~75 µm)	UV ageing (4 d)	Ciprofloxacin (CIP)	24 h	Aged PVC: sorption ↑ to pH 9 then ↓; aged PS peaked at pH 8	High salinity (35% *w*/*w*) reduced sorption by ~75% (PS) and ~86% (PVC).	Not studied (room T)	Not studied	Ageing improved sorption stability under unfavourable pH	Freundlich: PS-MPs-CIP: K_F_ = 0.54; n = 1.17 PVC-MPs-CIP: K_F_ = 0.74; n = 1.44 PSO: PS-MPs-CIP: k_2_ = 0.061; qₑ = 5.48 mg/g PVC-MPs-CIP: k_2_ = 0.296; qₑ = 3.28 mg/g
Zhang et al., 2018 [133]	PS foam (0.45–1 mm)	Natural beach weathering	Oxytetracycline (OTC)	54 h	pH 2–10; maximum sorption at pH ~5, decreasing at higher pH	0.15 M NaCl decreased sorption by ~30%.	Not studied (room T)	HA strongly increased sorption; FA only minor effect → DOM quality governs sorption	Weathering increased sorption ~2×	Freundlich: PS-MPs-OTC: K_F_ = 894 ± 84; n = 1.33 ± 0.03 Intraparticle diffusion (two-segment curve) PS-MPs-OTC: Segment1: k_1_ = 202, C_1_ = 154; Segment2: k_2_ = 531, C_2_ = 446
García-Pimentel, M.M et al., 2025 [118]	HDPE fragments (4–6 µm)	Marine weathering	TCS, methyl-chlorpyrifos (m-CPF)	24 h	Not reported	Fixed salinity: 38.5 PSU ≈ 0.77 M ionic strength.	15 °C vs. 25 °C; TCS sorption higher at 15 °C; m-CPF unaffected	Not studied	Weathering + temperature increased sorption for TCS	PSO: HDPE-MPs-TCS: k_2_ = 5.70 × 10^5^ g mg^−1^ h^−1^; qₑ = 3.226 × 10^−5^ mg/gm- HDPE-MPs-m-CPF: k_2_ = 5.90 × 10^5^ g mg^−1^ h^−1^; qₑ = 1.408 × 10^−5^ mg/g
Udenby et al., 2022 [134]	PE-MPs (200 µm; 1090 µm)	Hydrolytic + photo-oxidative (24 wks)	FLN, PHE	21 and 14 d	pH fixed (7.0 ± 0.2). No pH effect studied	Not studied.	Not studied (room T)	Not studied	Phenanthrene sorption ↑202% (200 µm) and ↑53% (1090 µm). Fluoranthene ↑11% (AFW), ↑64% (lake), ↓34% (1090 µm)	Langmuir tested but poor fit (R^2^ = 0.482–0.951). PSO validated only for virgin MPs, not weathered

## Data Availability

No new data were created or analyzed in this study. Data sharing is not applicable to this article.
